# Concrete-to-concrete shear friction behavior under cyclic loading: experimental investigation

**DOI:** 10.1038/s41598-022-13530-5

**Published:** 2022-06-08

**Authors:** Mohamad Taklas, Moussa Leblouba, Samer Barakat, Ahmed Fageeri, Firass Mohamad

**Affiliations:** Department of Civil and Environmental Engineering, College of Engineering, University of Sharjah, P.O. BOX 27272, University City, Sharjah, United Arab Emirates

**Keywords:** Engineering, Structural materials

## Abstract

This study investigated the concrete-to-concrete friction behavior under dynamic cyclic loading at different loading rates, vertical loads, and surface roughness. The present work answers essential questions about the dynamic behavior of concrete-to-concrete friction since most of the available literature deals with static or quasi-static loading conditions. To this end, an experimental program was devised by casting 96 concrete blocks. A total of 48 dynamic push–pull tests were performed on each pair of blocks (mobile top block and fixed bottom block). Test variables included three types of surface roughness, four different loading rates, and two normal stresses. Performance measures included the static and dynamic friction forces coefficients of static and kinetic friction in addition to effective stiffness and effective damping. Moreover, the test results showed that the static and kinetic friction coefficients, effective stiffness, and effective damping decrease with increasing loading rates. Moreover, increasing the normal stress increases the friction force, thus increasing the effective stiffness and reducing the effective damping surface for all surface roughness types. The effects of test variables on the hysteresis behavior were also investigated.

## Introduction

The friction behavior at the interface between two concrete blocks is an essential phenomenon in the design of concrete structures, especially structural members subjected to excessive shear loads. Notably, in reinforced concrete structural members at the stage after the failure of the interface, composite action no longer exists. At this stage, the friction between the two concrete parts should maintain a residual shear strength that, if quantified accurately, would help to avoid catastrophic failures of the concrete structures^1^.

**Figure 1:**
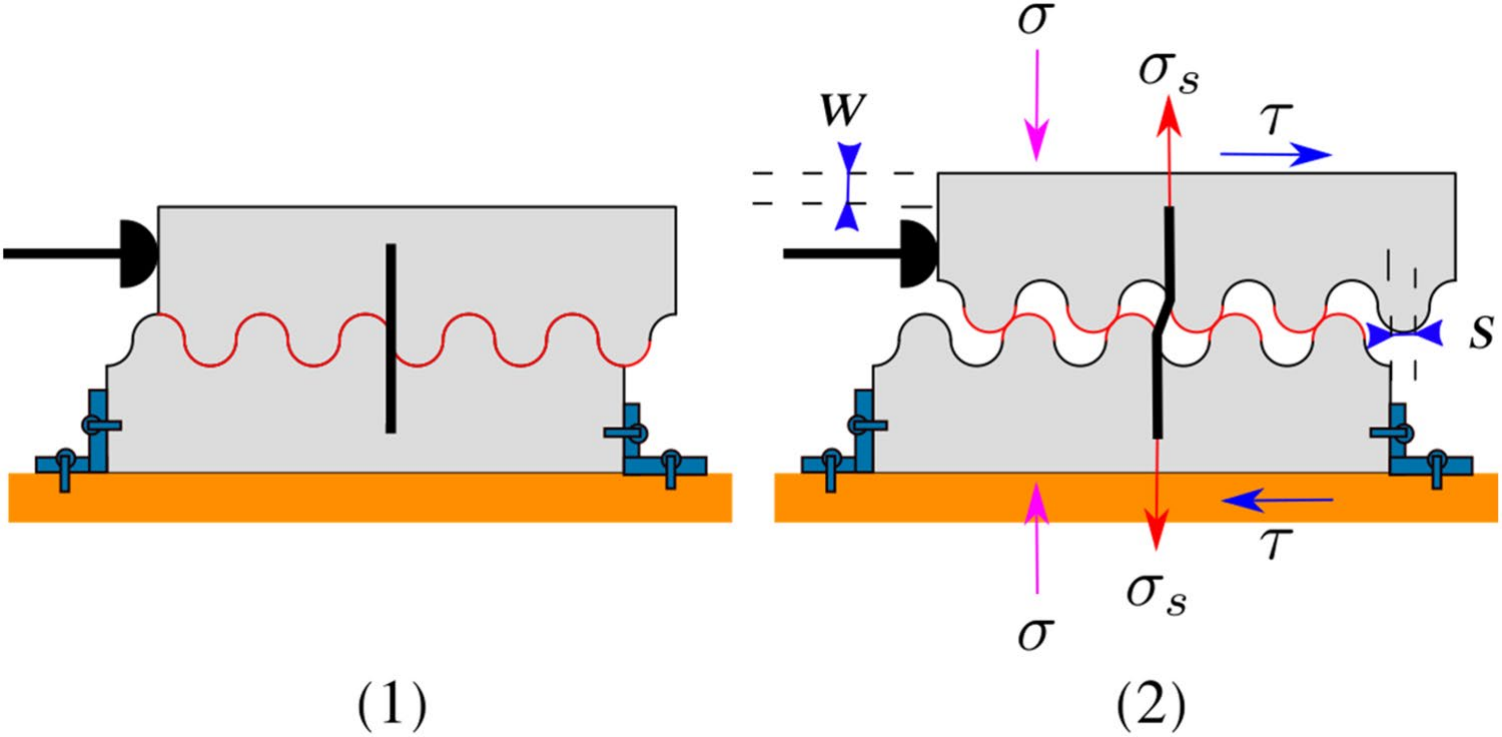
Saw-tooth model.

Generally, the shear-friction theory is used to study the friction between two concrete elements. The theory, which is governed only by friction, supposes that the mechanism of transferring the shear forces at concrete interfaces undergoes compression and shear forces simultaneously^2^. The saw-tooth model is commonly used to illustrate the fundamental concept of this theory^2^ (see Fig. 1).

This model considers the effect of normal forces applied to the shear plane and the reinforcement placed cutting across the interface. The shear-friction theory can be utilized to determine the shear capacity of various kinds of concrete interfaces, such as the interface between (a) two concrete elements cast at different periods; (b) support and concrete elements; and (c) two portions of concrete elements resulting from a crack^2^. Additionally, the theory is only applicable to the case where the interfacial behavior is governed by friction, dowel action, and cohesion in which slip exists at the interface between concrete layers relative to each other^2^.

Shear friction theory (SF) was first proposed by Birkeland and Birkeland^3^. They stated that the shear stress in rough surfaces produces tensile stresses in the reinforcement crossing the interface, resulting in compression in the joint. Also, the study compared the predicted ultimate shear stress, using shear friction theory, with the experimental results collected from previous studies and concluded that the theory successfully predicts the maximum shear stress at concrete interfaces. In addition, the shear friction theory was used to design knife and bearing connections, which were effectively used in structures, and concluded that the theory is also helpful in the design of connections. After this, Mattock and Hawkins^4^ proposed a modified shear friction theory (MSF) which included a term to account for the adhesion at the concrete interfaces. The ACI approach is partly based on this theory. Later on, the extended shear friction theory (ESF) was considered by the CEB-*fib* Model Code 2010^5^. This theory evaluated the critical effect of adhesion, friction, aggregate interlock, and dowel action mechanisms and captured their relationship by introducing interaction factors^6^.

The current study reports the results of an experimental program conducted to investigate the behavior of the concrete-to-concrete friction and the capacity at the interface between two concrete blocks cast at different times under cyclic loading. This test represents the case after the failure of the interface. The composite action is no longer established at the concrete-to-concrete interface, and the resistance is governed only by friction. In particular, the effects of the test rate, normal load, and surface roughness type on the bond strength and shear capacity are investigated.

## Review of related previous studies

The friction behavior at the concrete-to-concrete interface has been the subject of many studies to understand the mechanisms of shear capacity and the contribution of each mechanism in each stage. For instance, Zilch and Reinecke^7^ performed static push-off tests to investigate the shear friction at the interface between high-strength precast elements and normal-strength cast-in-place concrete blocks. The authors showed that the shear capacity at concrete interfaces could be divided into three mechanisms: friction, dowel action, and adhesion explained in more detail in^8^. Adhesion occurs due to the chemical bond at the interface between the concrete elements cast at different times. While an extreme strength is attained, debonding occurs at the interface between concrete elements. When a compression force is applied to the interface, the shear forces will be transmitted by friction. While the concrete elements are increasingly displaced relative to each other, the dowels that cut across the interface will be under tension, and yielding may occur. Hence, the dowels will exert compression force, and the shear forces will be transmitted by friction^7,8^.

Additionally, Xia et al.^9^ conducted direct shear tests on concrete interfaces while considering the normal stress, dowels, and coupling effect. The authors concluded that adding reinforcement and normal load increases the direct shear resistance and changes the behavior from brittle to ductile^9^; this is in line with the ESF theory that distinguishes between brittle (adhesive bond–based) and more ductile (addition of reinforcement) behavior^6^. It was also concluded that the friction, cohesion, and reinforcement action ratios are identical at the final residual stage^9^.

Many studies have been performed to study the behavior of reinforced concrete (RC) structures, with concrete-to-concrete interfaces, under cyclic loading. For example, Cavaco et al.^10^ tested the behavior of RC beams, with concrete interfaces located in the hogging part, under cyclic loading with increasing amplitude. The study concluded that casting interfaces have no effect on the strength of the structural member but can reduce the ductility of the structural member. In addition, it is concluded that the reinforcement and the direction of the interface do not affect the behavior. Furthermore, Cavaco and Camara^11^ experimented with evaluating the behavior of concrete interfaces submitted to a combination of bending moment and shear. The authors concluded that the load transfer capacity is decreased at the interface because of the cracks initiated by the bending moment, which led to the partial reduction in ductility but did not affect the strength of the specimens. To accommodate for the ductility loss in RC structures at the concrete interfaces, many studies investigated special detailing at concrete-to-concrete interfaces to improve the ductility. One of these studies was by Cavaco et al.^12^, who tried different details to enhance the ductility at the concrete-to-concrete interfaces. This study concluded that providing web reinforcement, normal to the concrete interfaces, can improve the ductility by limiting the shear slippages. Another study by Sørensen et al.^13^ investigated the effectiveness of a new shear connection at concrete interfaces for precast RC shear walls. In this design, the interfaces between precast parts are indented and attached by grouting with mortar. Also, the interfaces are reinforced with U-bar loops, which are overlapped and parallel to the wall plane. According to the results of the push-off experiment, it is concluded that the ductility is significantly improved using the new connection compared with the typical shear connection.

Based on previous major studies that evaluated the behavior of shear friction at the concrete interfaces, design codes^14,15^ presented some formulas to design concrete-to-concrete interfaces^2^. The CEB-*fib* Model Code 2010^5^ includes design recommendations for rigid and non-rigid bonds at concrete interfaces based on recent research^2^. Randl^6^ clarified the theory behind this and concluded that considering the recommendations based on bond type, along with the detailing requirements, forms the design of the concrete-to-concrete interface, taking into account the different mechanisms at the interface.

To understand the behavior of concrete parts sliding relative to each other, it is necessary to consider several variables, such as the surface finishing, loading rate, and normal stresses. Studying the concrete-to-concrete friction behavior under different test variables allows us to predict the shear capacity with better accuracy.

The surface roughness affects the strength of the bond, cohesion, and friction at the interface between concrete parts^16,17^. To determine the shear capacity at the concrete-to-concrete interface, design codes such as ACI 318^15^, Eurocode 2^14^, and CEB-*fib* Model Code 2010^5^ recommend different approaches to characterize the degree/level of roughness.

Concerning the surface roughness effect, several studies were conducted to quantify the correlation between the roughness of concrete surfaces and the strength of the bond between concrete parts cast at different periods. These studies included experiments to study the concrete-to-concrete friction behavior under static loading. For instance, Silva et al.^16^ carried out an experimental program to examine the shear capacity at the concrete interface with multiple surface roughness types of the concrete surface. The concrete surface was prepared by chipping with a jackhammer, wire-brushing, sand-blasting, and left as-cast using a formwork made out of steel. Additionally, pull-off experiments were performed to examine the bond strength under tension. From these results, it was concluded that the extreme strength value of the bond was obtained with sand-blasting and prewetting, and the substrate surface does affect the strength of the bond. Another study performed by Santos et al.^17^ investigated the possibility of quantifying the roughness of the substrate surface and correlating it with the interface bond strength. The results demonstrated that the roughness parameters are linearly correlated with the bond strength and that surfaces with higher average roughness values have higher bond strength.

Previous experimental studies on the concrete-to-concrete friction behavior, which included tests under dynamic loading conditions, investigated the effect of different test variables on the interface’s capacity between concrete surfaces. For instance, Tassios and Vintzeleou^18^ investigated the frictional behavior of plain concrete interfaces through small-scale monotonic and cyclic displacement-controlled laboratory tests. Their investigation considered the roughness and normal loads as test variables. The effect of these variables on the friction coefficient and shear capacity at the interface was investigated. The results showed that the shear capacity decreased due to surface grinding and that the coefficient of friction decreased under dynamic loading conditions. Fronteddu et al.^19^ conducted a shear test on concrete lift joint specimens with various surface roughness types to compare sliding joints’ static and dynamic behavior. Their test results showed that the coefficient of friction decreases with increasing normal load under both dynamic and static shear forces and that the shear strength depends on the surface roughness.

Previous dynamic tests also include the work of Figueira et al.^20^. They experimentally studied the shear behavior at the interface between a cast-in-place slab and a precast beam under monotonic and dynamic loadings with variable and constant amplitudes. High amplitude loading was the leading cause of interface failure due to increased cracking and concrete cover separation. However, low amplitude dynamic loading was shown to lead to failure in the steel bar because of fatigue as the shear strength decreased sharply. However, no evident damage was noted in the concrete during the tests.

## Problem statement and research objectives

Most studies on concrete-to-concrete friction behavior are based on static and quasi-static loading conditions. Experimental studies investigating the behavior of concrete-to-concrete friction under cyclic loading with different concrete surface roughnesses are scarce^18,19,21^. These include the work of Tassios and Vintzeleou^18^, which investigated the friction behavior of interfaces under monotonic and large reversed displacements, and that of Fronteddu et al.^19^. They also examined the friction behavior under static and dynamic loading. However, neither of these studies assessed the variation in friction force under the effect of different loading rates. Furthermore, only a few published experimental-based studies in this field investigated the behavior of concrete-to-concrete friction after the failure of the shear connection and its implications on the horizontal shear strength of the system, such as the one done by Leblouba et al.^1^. They investigated the behavior of the connection between precast concrete deck panels and concrete girders but did not consider different surface roughness types.

The damping of reinforced concrete structures is a complex phenomenon. It is approximated in the range of 2–5% of the critical damping based on the observed overall behavior of structures. It is known that the friction in cracked sections contributes to the bulk of this damping. Therefore, characterization of the friction at the concrete-to-concrete interface is of paramount importance towards a systematic and objective quantification of damping in reinforced concrete structures.

Considering the above, the objectives of this research are to:

1. Conduct dynamic cyclic push–pull tests on concrete block specimens and study the concrete-to-concrete friction behavior.
2. Assess the effect of concrete surface roughness on the behavior of horizontal shear strength at the interface.
3. Study the effect of loading test rate on the behavior of the horizontal shear strength at the concrete-to-concrete interface.
4. Study the effect of additional normal stresses on the horizontal shear strength at the concrete-to-concrete interface was studied.
5. Investigate the variation of static and kinetic friction coefficients, effective stiffness, and effective damping with the loading test rate, surface roughness, and normal stress.

## Experimental program

In this section, the experimental work is presented. In total, 48 dynamic push–pull tests were carried out to investigate the friction behavior of the concrete-to-concrete interface. In what follows, the geometry and mechanical properties of the test specimens are presented along with all test variables.

### Test specimens

**Figure 2:**
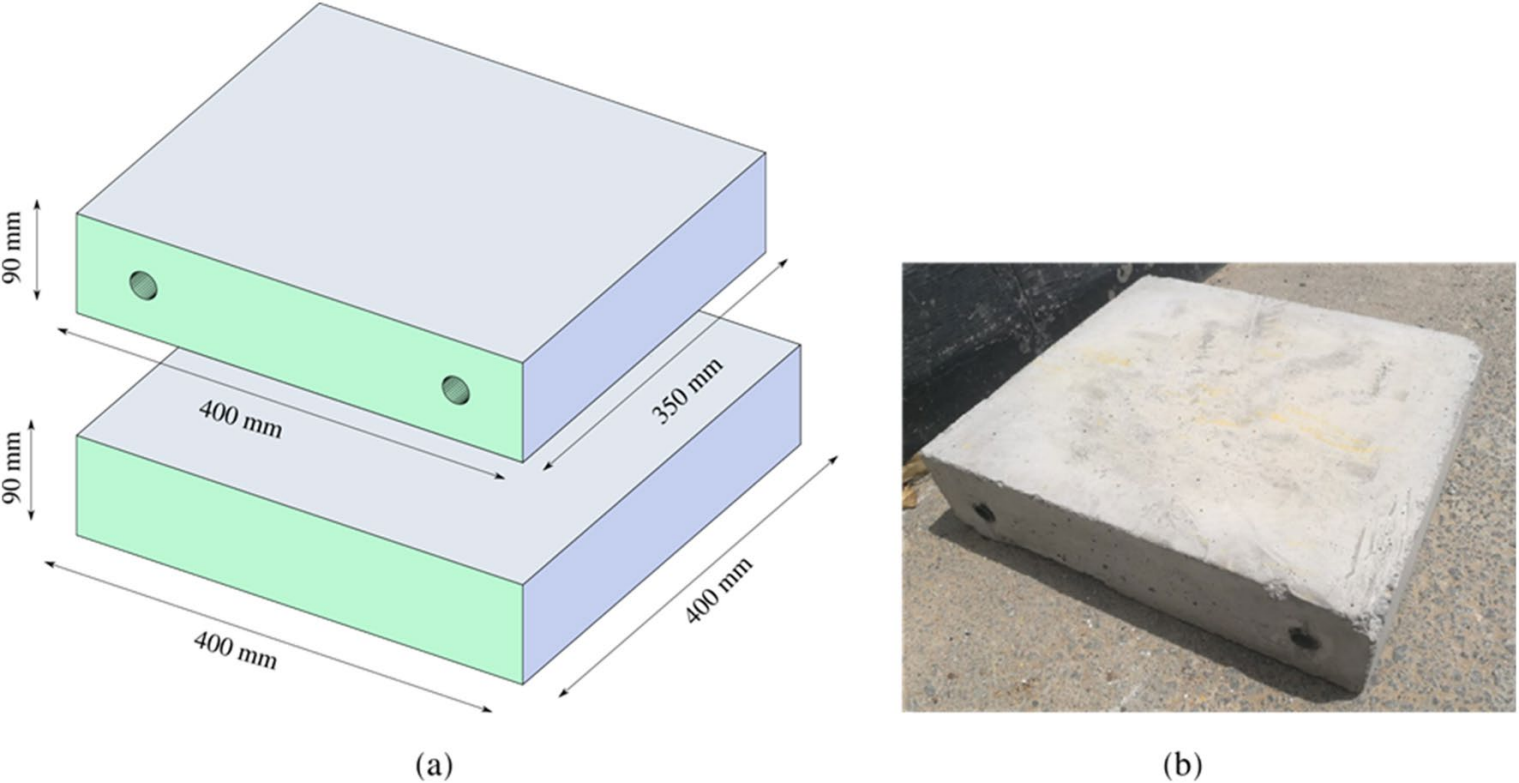
(**a**) Dimensions of test specimens and (**b**) concrete top block specimen.

Each test requires a pair of concrete blocks, one at the top, the moving part, and one at the bottom, the locked-in-place part. The geometry and dimensions of each block are presented in Fig. 2. (a) Dimensions of test specimens and (b) concrete top block specimen.

The top block has 400 × 350 × 90 mm dimensions with two holes for the long bolts that hold the block to the actuator’s head. The fixed bottom block has dimensions of 400 × 400 × 90 mm.

**Figure 3:**
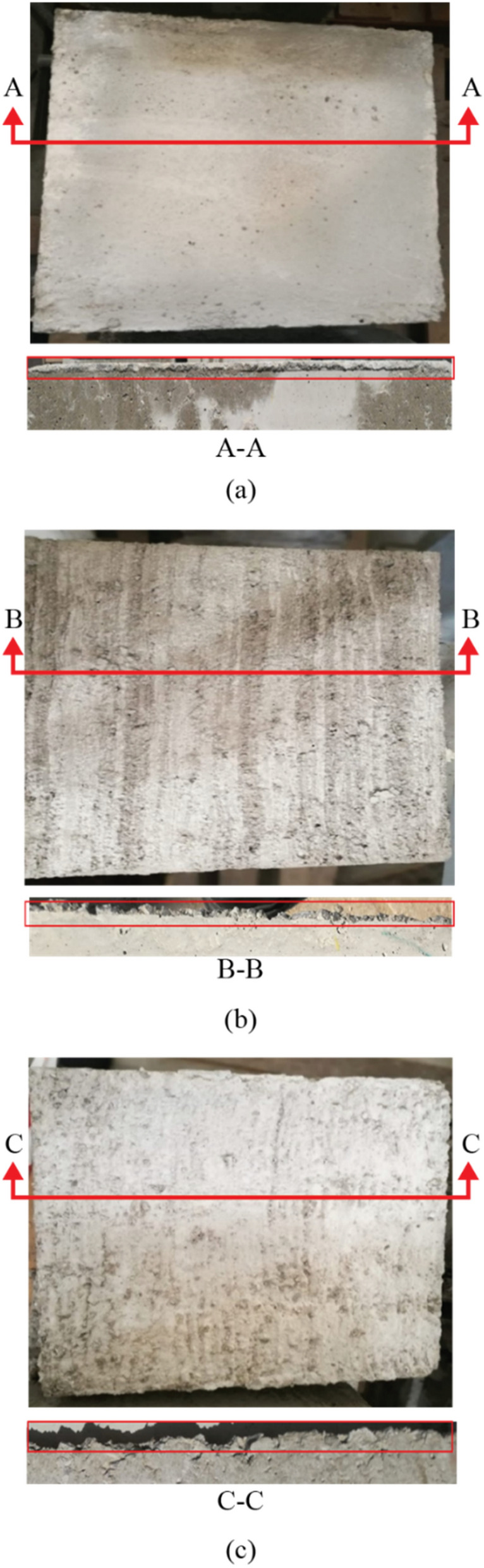
Concrete specimens with (**a**) smooth (as-cast), (**b**) medium rough (wire-brushed), and (**c**) rough (raked) surface roughness.

The bottom surface of the top moving block was left as-cast, i.e., the surface was not disturbed. However, the top surface of the bottom fixed block was altered to achieve three types of surface roughness: smooth (i.e., as cast), medium rough, and rough (achieved by raking the surface with a trowel). A smooth surface is achieved by finishing the surface with a typical finishing trowel, as shown in Fig. 3a, which is the conventional method of casting and finishing. On the other hand, a medium rough surface was achieved by wire-brushing the surface before concrete hardening. The brushing of the concrete surface was performed in one direction only, as shown in Fig. 3b. The parallel troughs were perpendicular to the direction of the motion. The specimen’s surface was raked with a trowel that had 6 mm-deep grooves for the rough surface. The grooves were inserted into the concrete surface at one edge and then moved along the whole surface so that the surface would be roughened in one direction, similar to the medium rough specimens (Fig. 3c).

As mentioned earlier, 48 tests were run using 96 concrete blocks (i.e., each test required one pair of blocks). The test variables included the test rate, normal stress, and surface level of roughness. As reported in Table 1, there are four loading rates (2, 5, 10 and 20 mm/s), two additional normal stresses (0 MPa and 0.25 MPa), and three roughness levels (S: smooth, M: medium rough, and R: rough). Note that there was always inherent normal stress due to the weight of the top moving block. Each test was repeated twice to ensure that all tests were reproducible with minimum discrepancy. There was no need for a third test since the discrepancy between every repeated test was acceptable. The test labels (column #2 in Table 1) consist of 1 letter followed by 3-digit numbers separated by dashes. The letter at the beginning represents the surface roughness (S, M, R). The next digit represents the test rate in mm/s, the normal stress in MPa, and finally, the test number (original or duplicate). For example, the label M-10-0.25-2 corresponds to a medium rough concrete surface, a test rate of 10 mm/s with an additional 0.25 MPa, and it is the duplicated (i.e., 2nd) test.

**Table 1:** Test notation and parameters.

Test #	Specimen	Surface roughness type	Sliding velocity (mm/s)	Normal stress (MPa)
1	S-2-0-1	Smooth (As-cast)	2	0
2	S-2-0-2		2	
3	S-5-0-1		5	
4	S-5-0-2		5	
5	S-10-0-1		10	
6	S-10-0-2		10	
7	S-20-0-1		20	
8	S-20-0-2		20	
9	S-2-0.25-1	Smooth (As-cast)	2	0.25
10	S-2-0.25-2		2	
11	S-5-0.25-1		5	
12	S-5-0.25-2		5	
13	S-10-0.25-1		10	
14	S-10-0.25-2		10	
15	S-20-0.25-1		20	
16	S-20-0.25-2		20	
17	M-2-0-1	Medium Rough (Wire-brushed)	2	0
18	M-2-0-2		2	
19	M-5-0-1		5	
20	M-5-0-2		5	
21	M-10-0-1		10	
22	M-10-0-2		10	
23	M-20-0-1		20	
24	M-20-0-2		20	
25	M-2-0.25-1	Medium Rough (Wire-brushed)	2	0.25
26	M-2-0.25-2		2	
27	M-5-0.25-1		5	
28	M-5-0.25-2		5	
29	M-10-0.25-1		10	
30	M-10-0.25-2		10	
31	M-20-0.25-1		20	
32	M-20-0.25-2		20	
33	R-2-0-1	Rough (Raked)	2	0
34	R-2-0-2		2	
35	R-5-0-1		5	
36	R-5-0-2		5	
37	R-10-0-1		10	
38	R-10-0-2		10	
39	R-20-0-1		20	
40	R-20-0-2		20	
41	R-2-0.25-1	Rough (Raked)	2	0.25
42	R-2-0.25-2		2	
43	R-5-0.25-1		5	
44	R-5-0.25-2		5	
45	R-10-0.25-1		10	
46	R-10-0.25-2		10	
47	R-20-0.25-1		20	
48	R-20-0.25-2		20	

### Materials

All test specimens were cast using ordinary concrete. The concrete mix proportions were designed to achieve a compressive strength of 40.0 MPa (see Table 2). Compressive strength tests were performed as per ASTM-C39^22^, while splitting tensile strength tests were performed as per ASTM—496^23^. At seven days, the average compressive strength was determined to be 31.0 MPa and at 28 days was 43.7 MPa. The average splitting tensile strength of the concrete was determined to be 3.1 MPa at seven days and 4.2 MPa at 28 days.

**Table 2:** Concrete mix design.

Material	Specific gravity (SSD)	Water Abs (%)	Density (kg/m^3^)	Proportion (%)
Cement	3.15	–	154.8	14.63
Water	1.00	–	260	24.58
10 mm Aggregate	2.68	0.3	362	34.22
5 mm Aggregate	2.67	0.6	156.4	15.64
0–1 mm Dune sand	2.66	0.3	74.2	7.01

### Test setup and instrumentations

**Figure 4:**
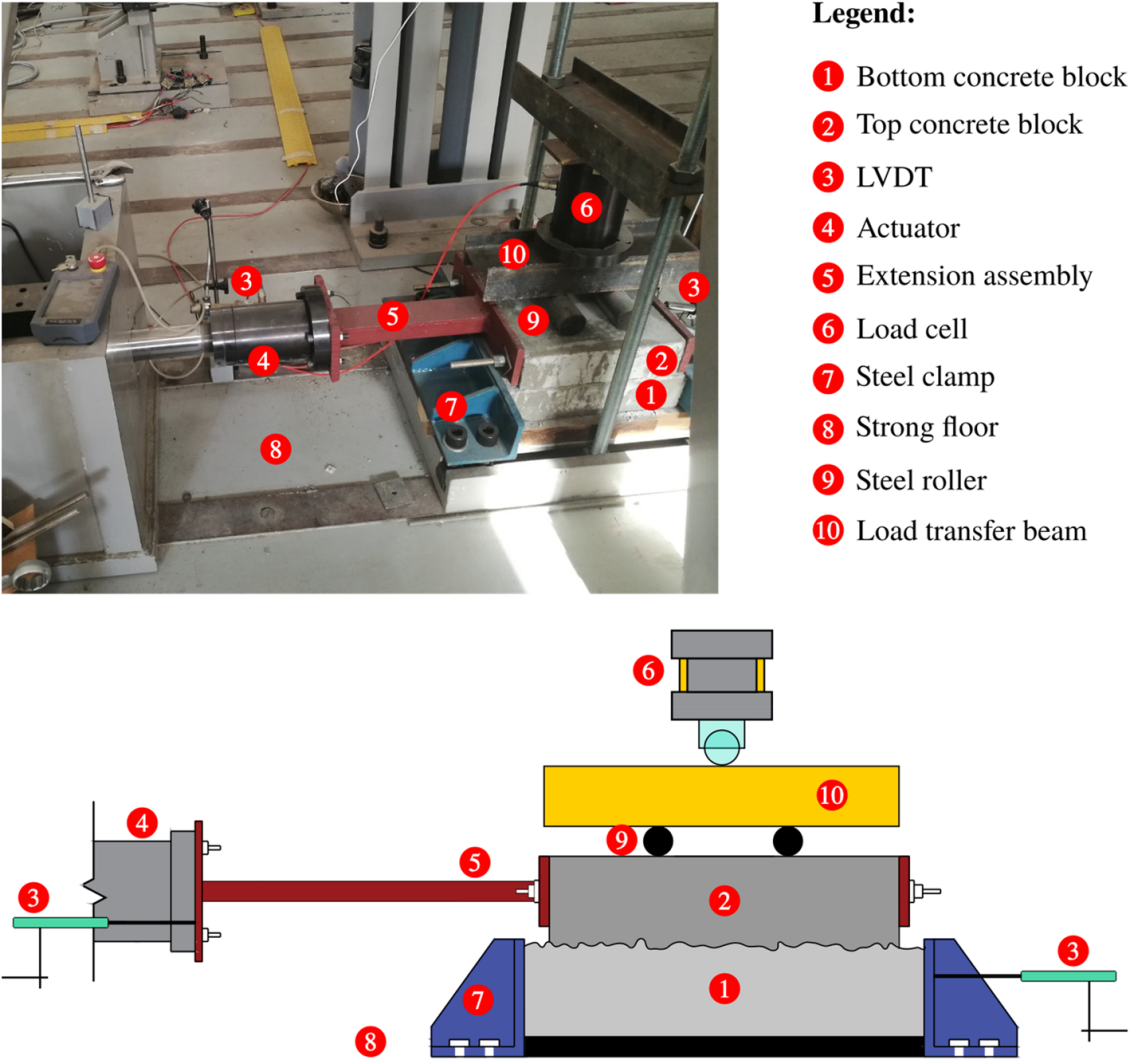
Test Setup and instrumentation.

The specimens were tested under a dynamic load applied by a 100-kN capacity Instron® actuator to a maximum displacement of ±20 mm. The normal load was applied using a high capacity load jack and transferred through a steel plate placed on two smooth steel cylinders on the top concrete block. The steel cylinders acting as rollers ensure the transfer of the vertical load to the concrete block and, at the same time, ensure the free horizontal movement of the top block relative to the bottom block. A custom-made U-shaped steel setup held the bottom concrete block to the strong floor. The top concrete block was connected to the horizontal actuator head from both sides (back and front) using steel plates and long bolts to transfer the exerted dynamic load and avoid overturning. The complete test setup is shown in Fig. 4.

For the instrumentation, two linear variable differential transformers (LVDTs) were used to ensure the integrity of the lab experiments. The first LVDT was attached to the actuator’s head for quality control of the experiment, while the second LVDT was attached behind the U-shaped steel set up to detect any movement of the bottom block, which was supposed to be fixed throughout the test.

## Results and discussion

The test results included the recorded actuator’s horizontal force, vertical force reaction, and horizontal displacement of the top concrete block. The friction force is assumed to be the same as the horizontal force since only friction resists the force without shear connectors or dowels. From the test results, several quantities were computed: the dynamic friction force ($f_{k}$), the static friction force ($f_{s}$), the normal load ($N$), and the normal stress, which was obtained by dividing the normal load by the area of the top concrete block (0.14 m^2^), the coefficient of dynamic/kinetic friction ($\mu_{k}$), the coefficient of static friction ($\mu_{s}$), effective stiffness ($K_{{{\text{eff}}}}$), energy dissipation per cycle ($W_{{}} D$), and effective damping ($\beta_{{{\text{eff}}}}$). Table 1 summarizes all computed quantities.

The effective stiffness $$k_{{{\text{eff}}}}$$ of the concrete blocks can be evaluated from the force–displacement curve in terms of the maximum ($$f^{ + } ,x^{ + }$$) and minimum ($$f^{ - } ,x^{ - } )$$ forces and displacements as:

1 $$ k{ }_{{{\text{eff}}}} = \frac{{f^{ + } - f^{ - } }}{{x^{ + } - x^{ - } }} $$

The effective damping $\beta_{{{\text{eff}}}}$ for ${\text{x}}_{{{\text{max}}}} \left( {\text{Maximum displacement}} \right){ } \ge {\text{x}}_{{\text{y}}} \left( {\text{Yield displacement}} \right)$ is defined as the area of the hysteresis loop, $W_{D}$, divided by $4\pi W_{S}$:

2 $$ \beta_{{{\text{eff}}}} = \frac{{W_{D} }}{{4\pi W_{S} }} $$

The area of the hysteresis loop $W_{D}$ is given by^24^:

3 $$ W_{D} = 4f_{k} \left( { x_{max} - x_{y} } \right) $$

and $W_{D}$ represents the energy dissipated per cycle, which is due mainly to the plastic deformation of the lead spring. The strain energy stored at maximum displacement, $W_{S}$, is given by^24^

4 $$ W_{S} = \frac{1}{2} k _{{{\text{eff}}}} x^{2}_{max} $$

The effective stiffness, effective damping, and energy dissipation calculated for all tests are presented in Table 3.

**Table 3:** Friction, stiffness, and damping quantities.

#	Specimen	$f_{s}$(kN)	$f_{k}$(kN)	$N$(kN)	$\mu_{s}$	$\mu_{k}$	$K_{{{\text{eff}}}}$(kN/m)	$W_{D}$(J)	$\beta_{{{\text{eff}}}}$(%)
1	S-2-0-1	2.38	1.29	2.49	0.956	0.518	64.5	90.0	55.5
2	S-2-0-2	2.05	1.27	2.73	0.751	0.464	63.3	92.7	58.2
3	S-5-0-1	1.48	0.75	2.79	0.531	0.269	37.5	50.7	53.8
4	S-5-0-2	1.97	0.86	3.76	0.524	0.229	43.0	61.8	57.2
5	S-10-0-1	2.23	0.84	3.23	0.69	0.26	42.0	57.6	54.5
6	S-10-0-2	2.6	1	4.1	0.634	0.244	50.0	69.7	55.4
7	S-20-0-1	1.6	0.77	2.96	0.541	0.26	38.5	57.2	59.1
8	S-20-0-2	2.3	0.75	3.47	0.663	0.216	37.5	53.6	56.8
9	S-2-0.25-1	23.6	13	35.28	0.669	0.368	650.2	782.1	47.9
10	S-2-0.25-2	21.7	17.05	35.42	0.613	0.481	852.6	934.3	43.6
11	S-5-0.25-1	23.8	18.9	35.17	0.677	0.537	945.0	1132.0	47.7
12	S-5-0.25-2	22.2	18.15	35.35	0.628	0.513	907.5	1091.9	47.9
13	S-10-0.25-1	26.3	17.79	35.26	0.746	0.504	889.7	1040.4	46.5
14	S-10-0.25-2	25.65	19.1	36.29	0.707	0.526	955.2	1059.7	44.1
15	S-20-0.25-1	23.4	12.5	35.2	0.665	0.355	625.1	696.0	44.3
16	S-20-0.25-2	23.8	14.67	35.43	0.672	0.414	733.7	906.6	49.2
17	M-2-0-1	1.48	0.81	1.33	1.109	0.607	40.5	55.2	54.2
18	M-2-0-2	1.11	0.63	1.03	1.08	0.613	31.5	43.3	54.7
19	M-5-0-1	0.98	0.69	1.17	0.839	0.591	34.5	48.9	56.3
20	M-5-0-2	1.26	0.78	1.31	0.959	0.594	39.0	52.7	53.8
21	M-10-0-1	1.19	0.9	1.52	0.783	0.593	45.0	63.7	56.3
22	M-10-0-2	0.84	0.6	0.91	0.924	0.66	30.0	44.4	58.8
23	M-20-0-1	0.84	0.7	1.34	0.628	0.524	35.0	47.6	54.1
24	M-20-0-2	1.1	0.69	1.24	0.886	0.552	34.3	46.6	54.1
25	M-2-0.25-1	25.96	19	35.29	0.736	0.538	950.2	1028.3	43.1
26	M-2-0.25-2	25.18	18.7	35.38	0.712	0.529	935.2	968.7	41.2
27	M-5-0.25-1	21.8	14.5	35.11	0.621	0.413	725.1	760.4	41.7
28	M-5-0.25-2	23	15.4	35.33	0.651	0.436	770.1	831.6	43
29	M-10-0.25-1	22.3	14.24	35.49	0.628	0.401	712.2	747.3	41.7
30	M-10-0.25-2	24.15	15.5	35.41	0.682	0.438	775.4	859.3	44.1
31	M-20-0.25-1	25.47	15.3	34.04	0.748	0.45	765.4	852.0	44.3
32	M-20-0.25-2	23	14.3	34.78	0.661	0.411	715.2	795.1	44.2
33	R-2-0-1	1.5	0.77	1.03	1.462	0.75	38.5	57.3	59.2
34	R-2-0-2	1.2	0.78	1.03	1.159	0.751	38.8	52.4	53.7
35	R-5-0-1	1.2	0.8	1.34	0.894	0.596	40.0	55.7	55.4
36	R-5-0-2	1.36	0.83	1.28	1.065	0.65	41.5	59.8	57.3
37	R-10-0-1	1.46	0.53	1.17	1.248	0.453	26.5	37.5	56.3
38	R-10-0-2	1.03	0.64	1.17	0.884	0.549	32.0	45.3	56.3
39	R-20-0-1	1.05	0.5	0.84	1.242	0.594	25.0	34.0	54.1
40	R-20-0-2	0.88	0.61	1.04	0.844	0.585	30.5	42.3	55.2
41	R-2-0.25-1	18.97	8.57	35.33	0.537	0.243	428.5	438.8	40.7
42	R-2-0.25-2	17.1	11	35.3	0.484	0.312	550.2	587.8	42.5
43	R-5-0.25-1	19.7	10.83	35.25	0.559	0.307	541.6	567.5	41.7
44	R-5-0.25-2	18.49	10.92	35.25	0.525	0.31	546.1	572.2	41.7
45	R-10-0.25-1	22	10.91	35.22	0.625	0.31	545.4	566.9	41.4
46	R-10-0.25-2	19.7	11	35.28	0.558	0.312	550.1	576.4	41.7
47	R-20-0.25-1	24.4	13.9	34.96	0.698	0.398	695.2	706.1	40.4
48	R-20-0.25-2	18.88	11.58	35.3	0.535	0.328	579.0	618.8	42.5

### Concrete surface condition after the test

**Figure 5:**
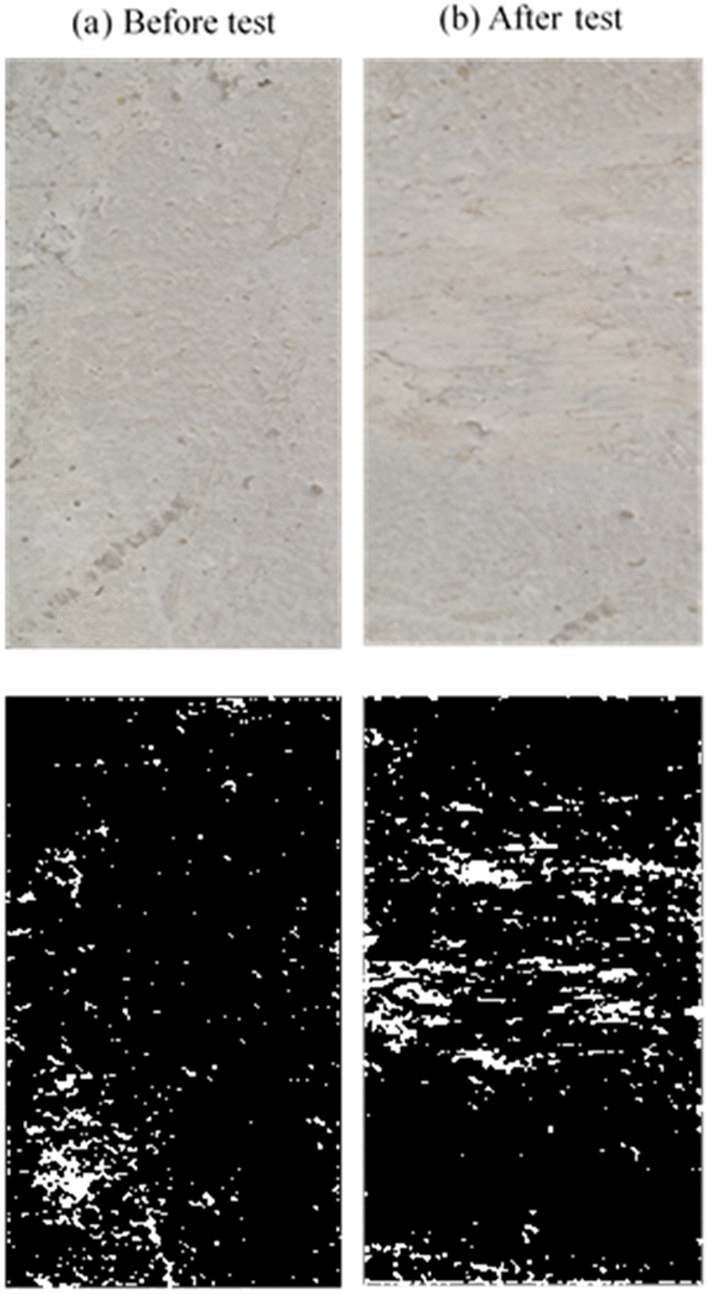
Smooth surface before and after test.

**Figure 6:**
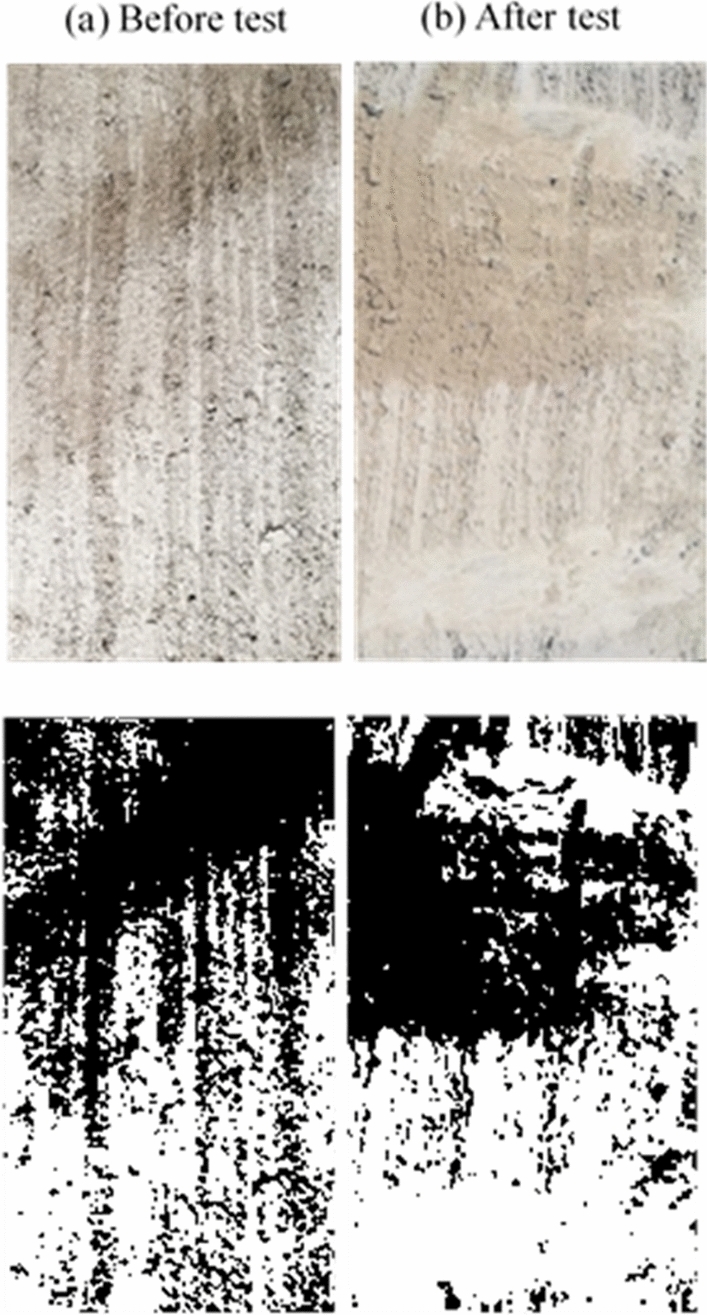
Medium rough surface before and after test.

**Figure 7:**
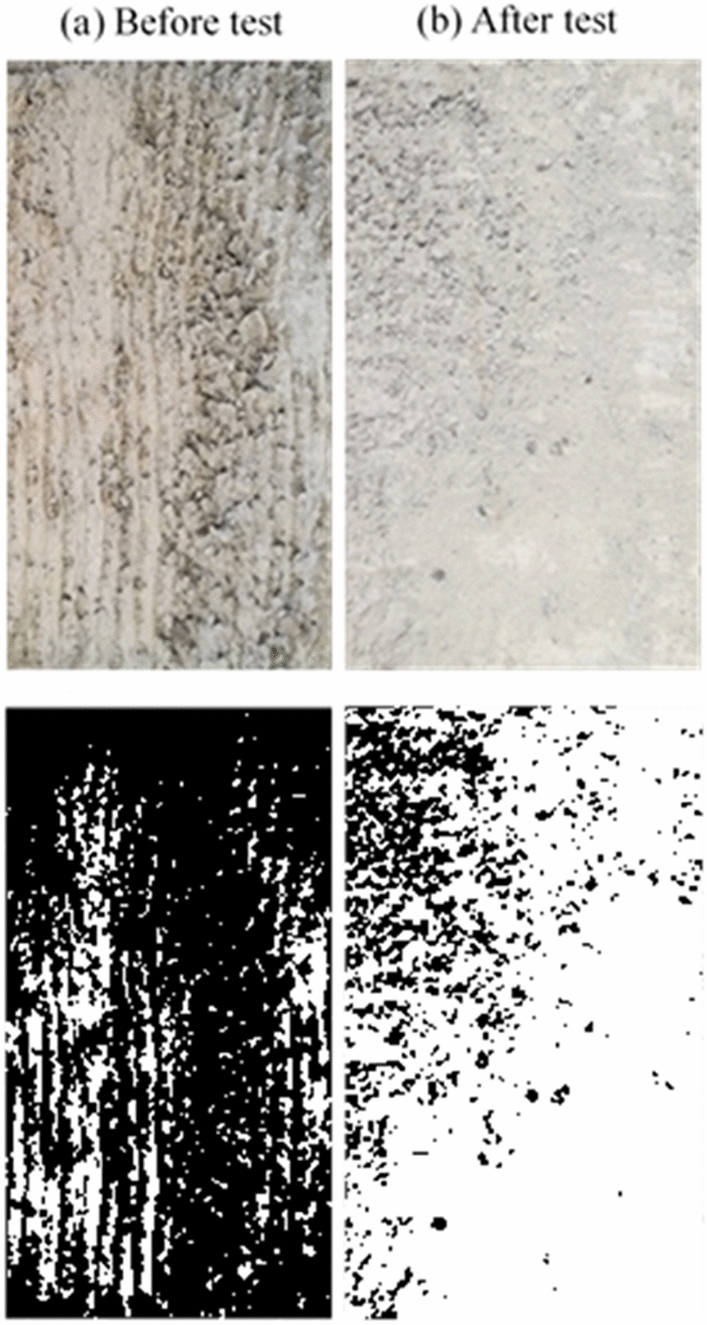
Rough surface before and after test.

After each set of tests, the concrete block’s surface was imaged and analyzed to determine the degree of grinding and damage. For the smooth (i.e., as-cast) surface, there was minimal grinding of the concrete surface after the test, as demonstrated in Fig. 5. Figure 5b shows that only minor scratches on the surface were visible after the test and without significant surface deterioration. The same observation was noticed by Tassios and Vintzeleou^18^, who concluded that there was no considerable deterioration of the smooth interfaces during testing. Moreover, small cracks were observed to develop at the surface edges when a high vertical force was applied to the surface. In medium rough and rough specimens, the concrete surface grinding after a test was more noticeable than in the case of as-cast samples, as shown in Figs. 5, 6 and 7.

Moreover, there was a clear cutoff of the surface asperities during the push–pull test in the rough specimens. This leads to the reasonable conclusion that increasing the average roughness of a surface leads to a greater surface deterioration after a push–pull test. In general, it is noted that increasing the test rate results in a higher surface deterioration in the medium rough and rough surfaces, while the deterioration at different loading rates for the smooth surface remains approximately the same. Also, images were binarized to estimate the ratio of white pixels to the total number of pixels to quantity and the difference in the state of surface deterioration before and after the test. After processing images after tests, the percentage of the white area to the total image area was 10.3% for the smooth surface concrete specimen, 57.6% for the medium rough, and 83% for the rough. These results indicate the level of surface grinding and deterioration experienced by concrete surfaces after tests depending on their original roughness level.

**Figure 8:**
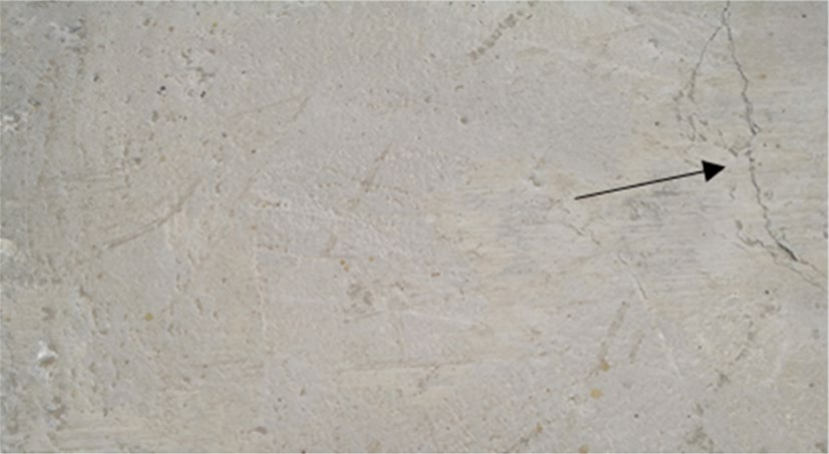
Diagonal cracks in a smooth specimen.

It is interesting to note that for some specimens, some cracks were developed at the edges under vertical stress of 0.25 MPa, as shown in Fig. 8. The reason behind this might be, as noted by Tassios and Vintzeleou^18^. They stated that for a shear stress value less than the one needed for the overriding of asperities, the shear resistance of the protruding asperities under high normal stress might be more than the tensile strength of the matrix. Therefore, a diagonal crack through the concrete matrix may occur before the overriding of the asperities, which will result in a premature failure mechanism. Moreover, Cavaco and Camara^11^ noted the diagonal cracks. They mentioned that this happens due to the decreasing friction capacity, which reduces the ability of a structural element to transfer shear loads.

### Load–displacement response

**Figure 9:**
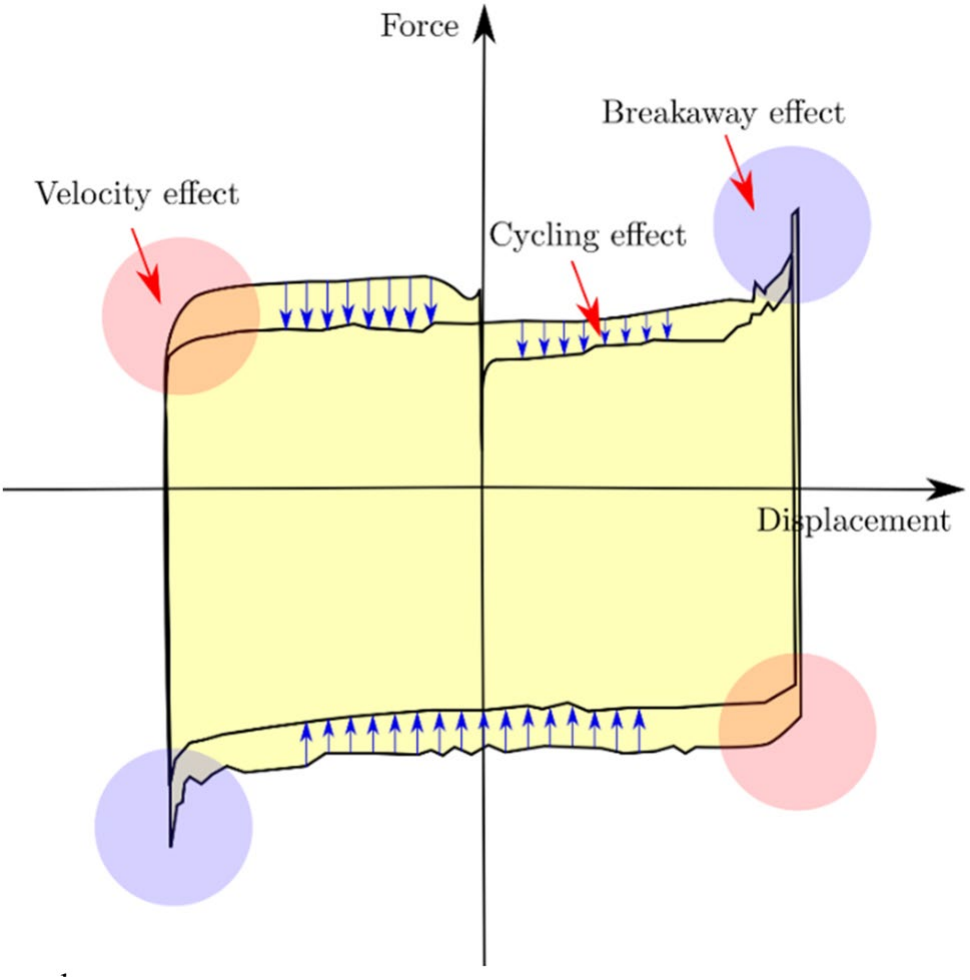
Typical load–displacement graph.

The frictional force and the applied vertical load are directly proportional according to the simplified Coulomb model in which the effect of sliding velocity is not considered. This assumption leads to a rectangular force–displacement hysteresis loop that differs from the typical experimental loop shape shown in Fig. 9. Analysis of the typical shape of the hysteresis loops indicates some major effects according to Lomiento et al.^25^:

- The breakaway effect before load reversal is represented by a rapid increase in the friction force at the beginning of the relative movement or each motion reversal, followed by a quick force release. The static friction condition and the stick–slip are the leading causes of this effect.
- The cycling effect is represented by the continued reduction of the friction force with the repetition of cycles.
- Figure 9 illustrates a 2nd loop narrower than the 1st loop, indicating deterioration of the friction force with an increasing number of cycles. This might be the grinding of the surface and the decrease in the height of the asperities.
- The velocity effect is represented by the friction force variation with the velocity. A speed reduction is expected to introduce lower values of friction force. This reduction can be noticed in the rounding of the shape of the loops when reaching the peak displacement.

**Figure 10:**
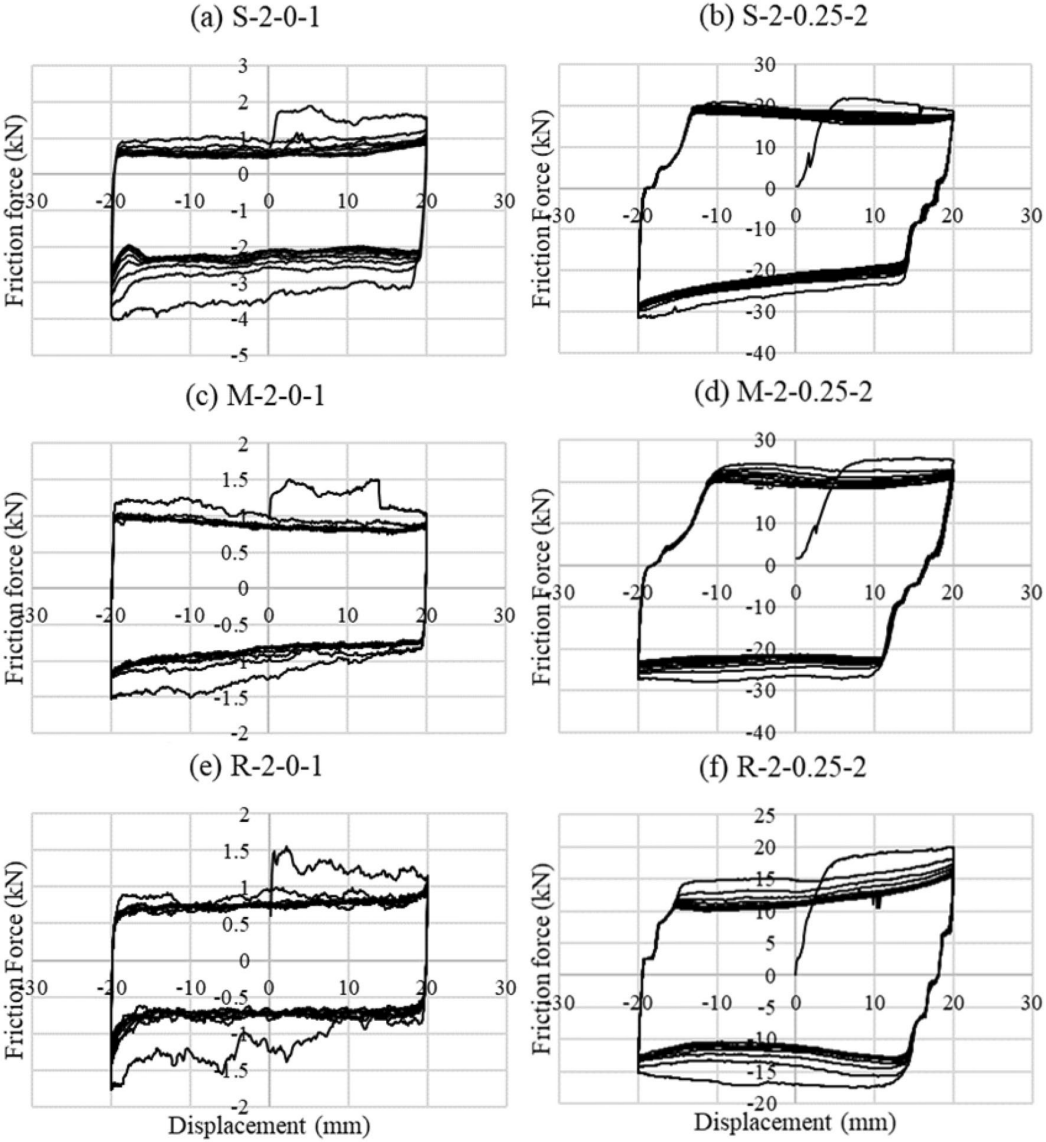
Friction force versus displacement graphs for tests with different vertical stresses.

**Figure 11:**
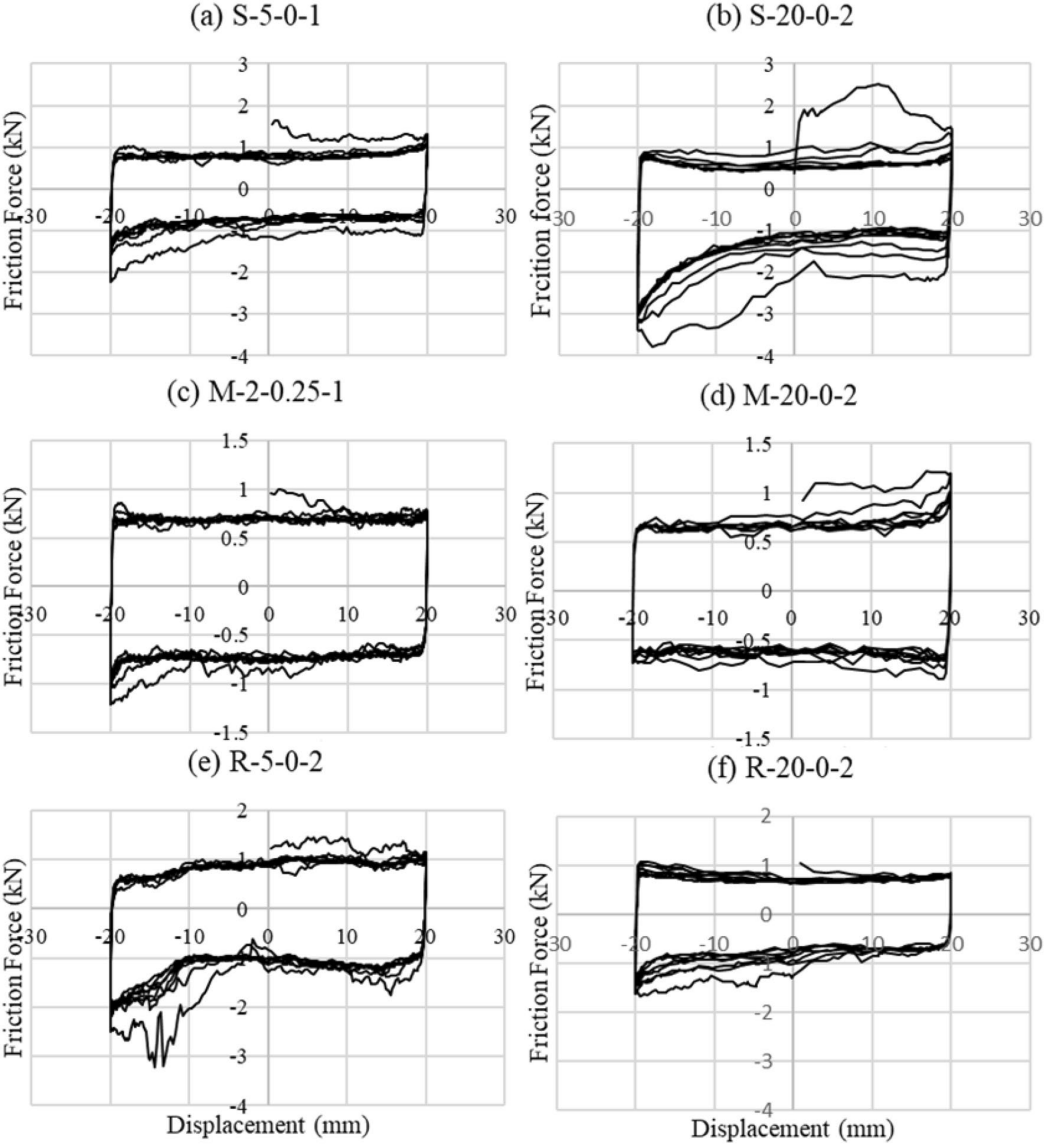
Friction force versus displacement graphs for tests with different loading rates.

The friction force versus the horizontal displacement curves for a subset of test specimens are shown in Figs. 10 and 11 to highlight the effects of vertical stress and test rate, respectively.

At the beginning of the first loading cycle, the horizontal load increases until the top block moves relative to the bottom block. This load represents the static friction, which is used to estimate the coefficient of static friction. This load decreases in subsequent loading cycles, indicating the kinetic friction phase at which the coefficient of kinetic friction occurs. It is important to note that the friction force is higher in the unloading phase of the first cycle than in the loading phase (see the unsymmetric first hysteresis loop in Fig. 10). This can be explained by the fact that upon unloading the specimen in the first cycle, the surface texture is almost intact in the reverse direction of the loading. Thus, the recorded increase in the friction force; however, in the subsequent loading, unloading, and reloading cycles, the surface texture remains the same due to the grinding after the first complete cycle. At this stage, the hysteresis loops are almost symmetric in all tests.

Moreover, and as expected, the friction force is higher in the tests with 0.25 MPa additional pressure. Generally, it is approximately 20 times higher than the friction force recorded in tests without extra pressure. This ratio is not sensitive to the surface roughness, as demonstrated in Fig. 10.

Another important observation is the rate of decrease of the friction force with each loading cycle and its connection to the surface roughness. The friction force diminishes slowly after each cycle in the smooth surface tests and remains the same (i.e., hysteresis loops overlap). The friction force decreases significantly after each cycle (i.e., hysteresis loops decrease in the area). The medium rough surface tests represent an average case where the friction force decreases rapidly but not as much as the rough surface tests. This is the case in most tests with 0.25 MPa additional pressure. This trend is due to the effect of the vertical pressure applied on the test specimens and its reduction rate for each surface roughness. As we will see later, the vertical force decreases rapidly and significantly with each loading cycle in rough surface tests but decreases slowly and slightly in smooth surface tests. Even though the shape of the hysteretic loops is different for different loading rates, the effect of the test rate on the strength of the test specimens is not noticeable (see Fig. 11).

### Load-velocity curves

**Figure 12:**
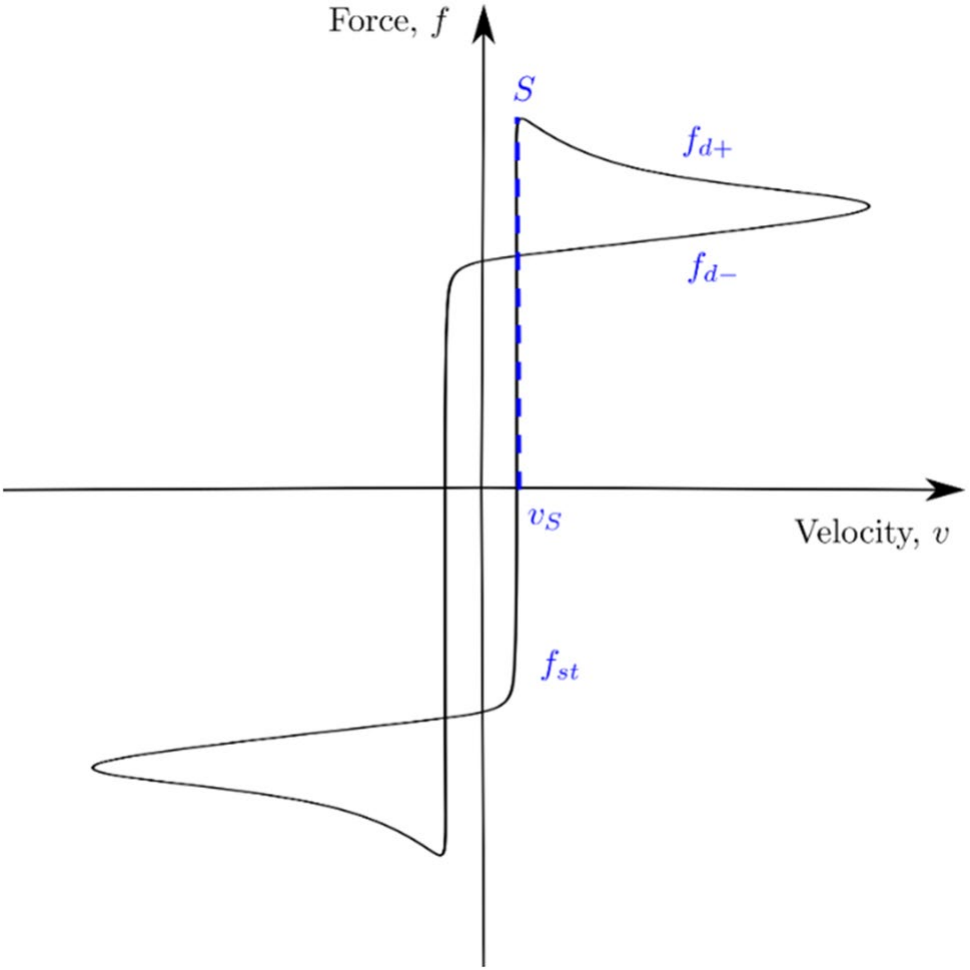
Typical load-velocity graph reproduced based on ref ^26^.

Previous experimental studies showed that the load-velocity graph follows the typical hysteresis shape shown in Fig. 12. $f_{st}$ in the graph represents the value of friction force at the presliding displacement, and $f_{d}^{ + }$ and $f_{d}^{ - }$ in the graph represent the values of friction force at the acceleration and deceleration phases, respectively.

The presliding displacement and the non-reversibility of the friction force are the most important phenomena that can describe the behavior of the load-velocity graph. The small hysteresis around the zero relative velocity describes the presliding motion^26^, and nonidentical slopes of friction force for the deceleration and acceleration phases characterize the non-reversibility^27^. Nonreversibility was noticed in many experiments (e.g.,^28^). The presliding displacement occurs due to the friction force’s spring-like behavior before the actual relative sliding or, in other words, due to the tangential stiffness at the contact interface between the bodies^29^.

**Figure 13:**
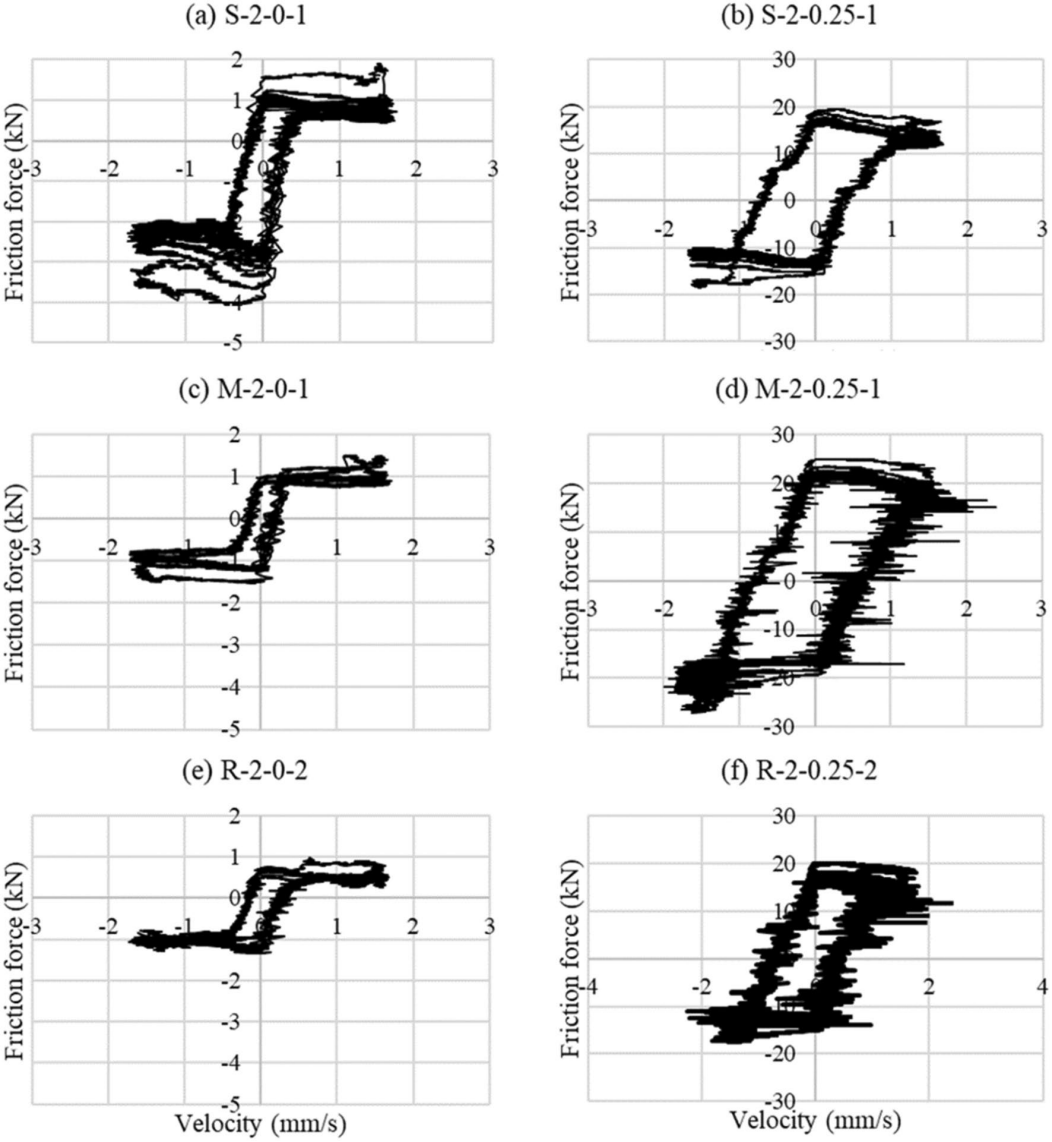
Friction force versus velocity graphs.

The general behavior of the load-velocity graph takes the shape of the letter S, as shown in Fig. 13. The same behavior was noticed by Fronteddu et al.^19^, in which the velocity starts from zero and then increases to reach a maximum value; then, the motion is reversed. It was observed that the S portion occurred when the upper concrete block slowed down for shear load reversal. As expected, increasing the vertical stress increases the hysteresis loop area since (as demonstrated in Sect. 5.2) increasing the vertical stress increases the friction force.

### Vertical load behavior

At the beginning of each test, two different vertical stresses were applied to the top concrete block 0 and 0.25 MPa. The load cell placed above the top concrete block monitored the vertical force during tests. The shear test configuration ensures that the normal stresses at the interface are free to vary with the horizontal shear displacement. Tassios and Vintzeleou^18^ investigated the concrete-to-concrete friction behavior under cyclic loading. In their tests, the authors maintained almost constant normal stress. However, it was noted that normal stresses also increase in real reinforced concrete structures when an interface is subjected to shear. Together with shear displacement and crack width, normal stresses are expected. Hence, the tests were performed under free normal stresses to represent a real scenario. It is clear that vertical load changes throughout the test. As specimens are loaded horizontally, the vertical load goes up and down depending on the magnitude of dilation and direction of displacement. In the beginning, the vertical force decreases. After the failure of the aggregate interlock mechanism at the concrete-to-concrete interface and as the shear displacement increases, the top block overrides the asperities and loose aggregates at the interface, which increases the tendency to cause dilation and increases the crack width. This, as a result, causes an increase in the normal load recorded by the vertical load cell since the specimen is restricted from moving in the vertical direction.

**Figure 14:**
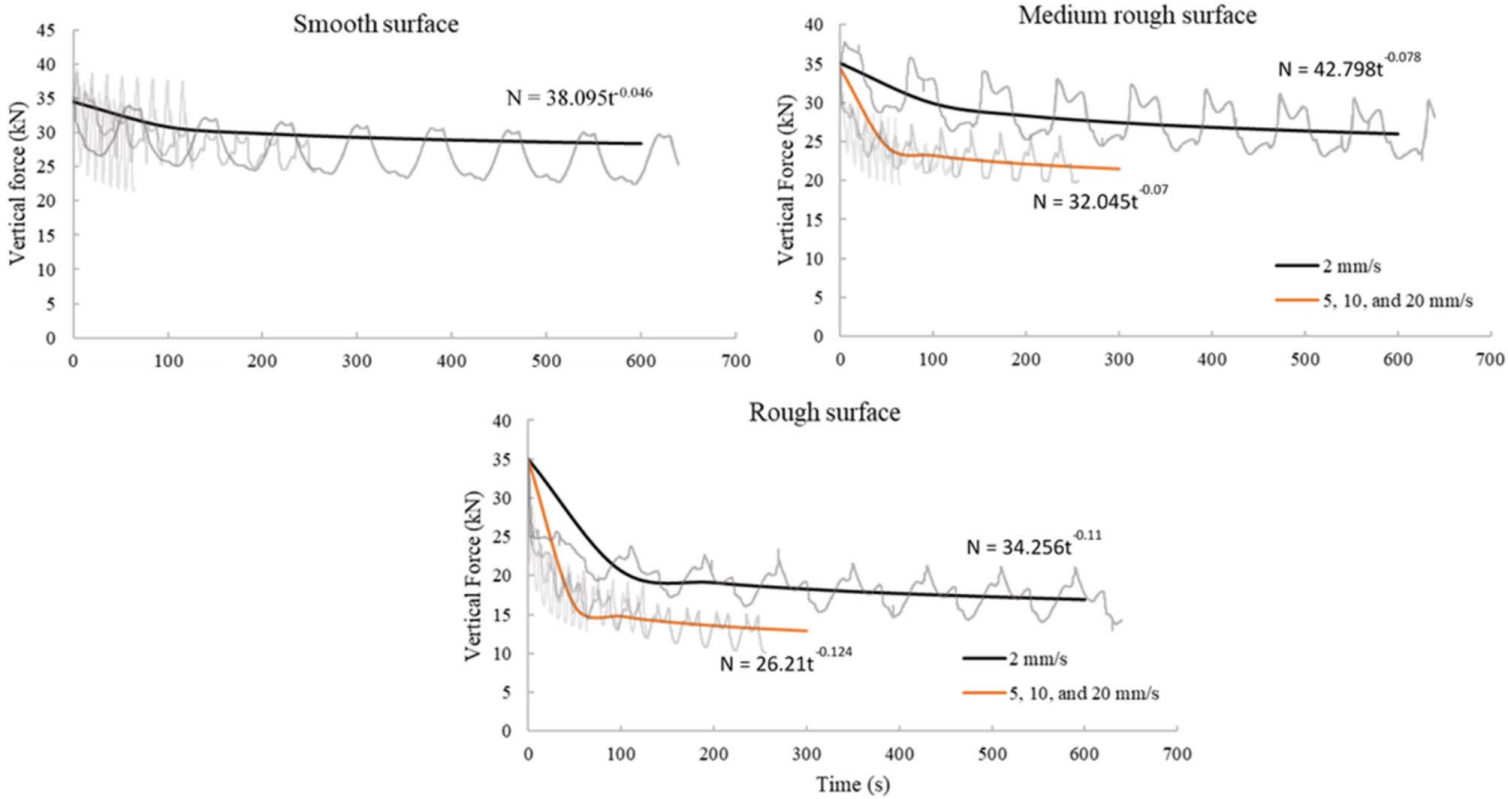
Vertical force trends.

Additionally, it is noted that increasing the test rate resulted in a greater reduction in the vertical force at the end of the test for both medium rough and rough surfaces. This agrees with the observation of higher degradation of medium rough and rough surfaces at higher loading rates, which will result in a reduction in the recorded vertical force. In general, it is noted that the decrease in the vertical force was the lowest for the smooth surface, taking into account all loading rates, and the reduction was higher for the medium rough and rough surfaces, as shown in Fig. 14. From this, it can be concluded that surfaces with higher average roughness values have higher vertical load degradation. In construction practice, rough surfaces are widely used since their static shear friction capacity is higher than smooth surfaces. Many studies investigated the bond strength at rough surfaces altered using different techniques^30,31^.

For the tests where vertical stress of 0.25 MPa is applied, all test specimens start with almost similar vertical forces regardless of the surface condition, approximately 35 kN (0.25 MPa over a cross-sectional area of the concrete block). However, this force decreases rapidly with the surface roughness. In smooth surface tests, the effect of the test rate is negligible; at all speeds, the vertical force reduces slowly with time. In medium rough, and rough surface tests, the effect of the test rate is substantial between a 2 mm/s test rate and 5 mm/s. However, above the 5 mm/s test rate (5, 10, 20 mm/s), the test rate has a negligible effect, especially in rough surface tests. This is illustrated in Fig. 14. This trend is not the same in the 0-MPa pressure tests; the decrease in the recorded vertical load is not significant after each cycle.

**Figure 15:**
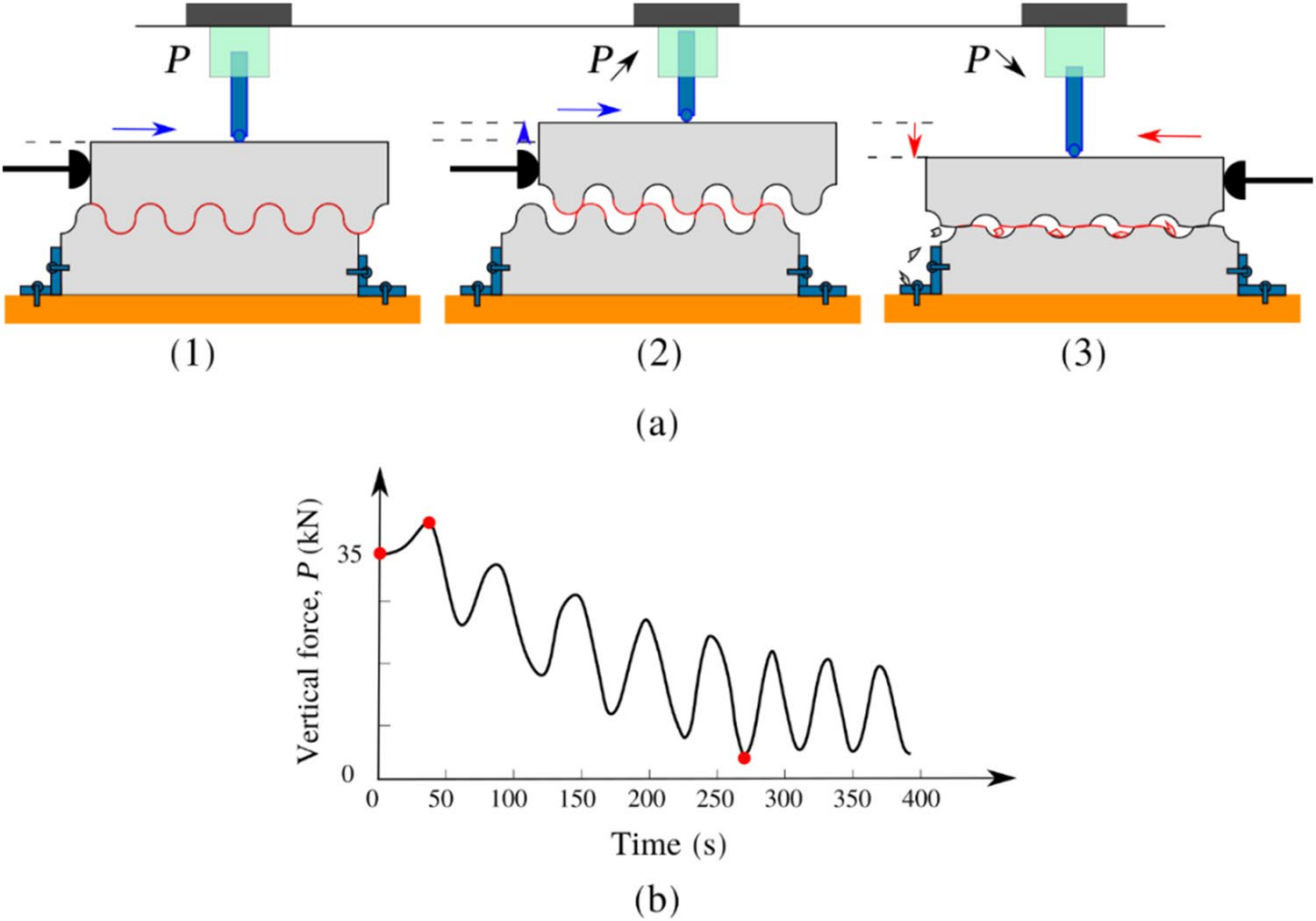
Vertical load behavior.

Usually, in a test involving a perfectly smooth surface, the vertical load should remain the same regardless of the test rate. However, in the case of rough surfaces with many asperities, the top block, when pushed, moves up, thus increasing the vertical force (reaction of the downward jack). During load reversal, some of the asperities are reduced in height (R_a_ smaller), and as a result, the vertical load decreases. This behavior continues with subsequent load cycles where the vertical load increases and decreases. However, the overall trend is that the vertical load decreases after each complete load cycle. This is illustrated in Fig. 15. This behavior of changing vertical stress can represent the case of deep underground scenarios where the constant normal stiffness (CNS) boundary condition is more applicable than the constant normal stress (CNL) condition since, in this case, the normal stress is not constant^32^.

## Parametric study

**Figure 16:**
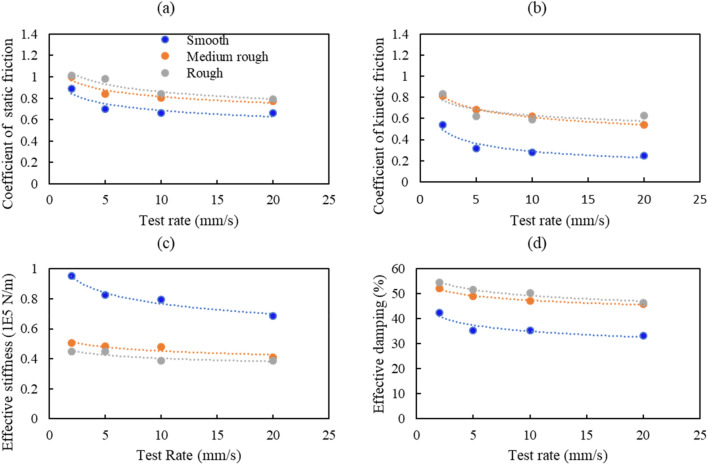
Variation of selected response quantities with the test rate at 0.0 MPa additional normal stress.

**Figure 17:**
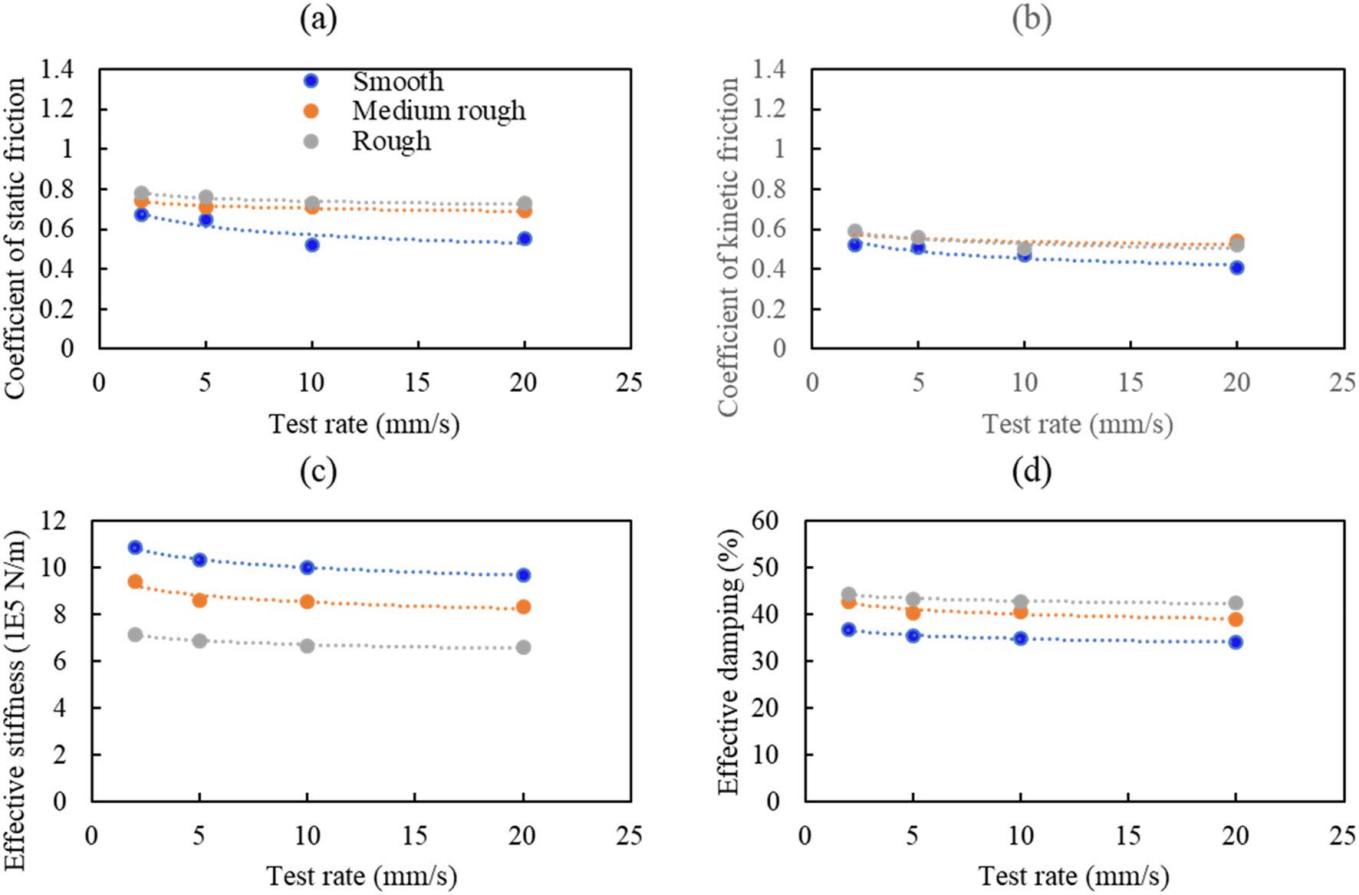
Variation of selected response quantities with the test rate at 0.25 MPa additional normal stress.

It is clear from the results that many parameters affect the concrete-to-concrete friction behavior in different ways. The response quantities discussed in this section include the coefficient of static friction, coefficient of kinetic friction, effective stiffness, and effective damping. The variation in these quantities (shown in Figs. 16 and 17) is investigated by considering the test rate, roughness, and normal stress effects.

### Effect of test rate and surface roughness

Experimental data for tests under 0.0 MPa and 0.25 MPa additional normal stress showed that, in general, increasing the test rate decreases the coefficients of static and kinetic friction, as shown in Figs. 16 and 17, for 0.0 and 0.25 MPa additional normal stress, respectively. Both coefficients have the highest value at the 2 mm/s test rate and the lowest at 20 mm/s. The decrease in the coefficient of friction at higher loading rates might be due to the “indentation creep” that low loading rates cause an increase in the actual contact area^33^. The static and kinetic friction coefficients are the highest in rough surface tests due to the grooves and asperities present on the concrete surface. The decrease in the static and kinetic friction coefficients was fast (between 21 and 26%) for tests performed under 0.0 MPa additional normal stress and slower (between 5 and 20%) for tests done under 0.25 MPa additional normal stress.

As shown in Fig. 16c and Fig. 17c, the variation of the effective stiffness and effective damping with test rate follows a similar trend to the coefficient of friction. This is in line with the findings of Leblouba et al.^1^. The effective stiffness is the highest in smooth surface tests due to the lower height of asperities than on rough surfaces. The decrease in effective stiffness was between 14 and 27% for tests performed under 0.0 MPa additional normal stress and 8–12% for tests performed under 0.0 MPa additional normal stress. Similarly, the effective damping (Fig. 16d and Fig. 17d) decreases with the increase in test rate, and this increase slows down at higher normal stresses.

In some cases, the kinetic coefficient of friction on a smooth surface is lower than in practical situations (e.g., Fig. 16b). This can be attributed to the significant uncertainties associated with the friction phenomenon and to the imperfection of the as-cast concrete surfaces, which in some specimens have some protruding asperities which cause an increase in the initial normal stress/force lower coefficient of friction.

The average coefficient of static friction varies between 0.5 and 0.9 for the as-cast surface and between 0.7 and 1.1 for wire-brushed and raked surfaces. Compared with CEB-*fib* Model Code 2010^5^, the experimental static coefficient of friction values for the as-cast surface in this study best matches the smooth surface range (0.5–0.7). Regarding wire-brushed and raked surfaces, the experimental static coefficient of friction values best fits the range of the rough surfaces (0.7–1.0). It can be noted that there are some differences between the experimental and CEB-*fib* Model code ranges due to many possible reasons. One of the reasons might be the imperfection of the concrete surfaces. The design codes should consider this by providing statistical parameters (e.g., mean, standard deviation, etc.) based on extensive experimental data rather than providing exact ranges. Also another reason might be the use of different techniques for roughening the concrete surfaces compared with the CEB-*fib* Model code. This can be the case in rough surfaces where the CEB-*fib* Model code mentioned roughening methods (sand-blasted and high-pressure water blasted) are different from those used in this study.

### Effect of normal stress on the friction force

**Figure 18:**
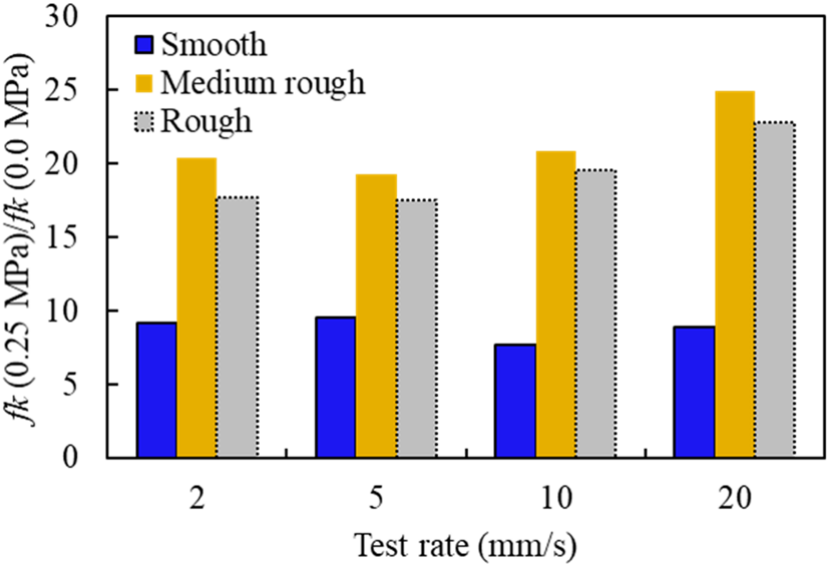
Variation in friction force with test rate for different concrete surface conditions.

As concluded previously, higher normal stress increases the friction force for all tests. Figure 18 shows the variation of the kinetic friction ratio $f_{k}^{{0.25 {\text{MPa}}}} /f_{k}^{{0.0 {\text{MPa}}}}$ with the test rate and for all concrete surface conditions. The increase in the friction force is more pronounced on the rough surfaces than on the smooth surface.

For instance, in rough surface tests, the ratio $f_{k}^{{0.25 {\text{MPa}}}} /f_{k}^{{0.0 {\text{MPa}}}}$ increases between 15 and 25, while in smooth surface tests, the ratio drops to 5 to 10.

## Summary and conclusions

In this paper, we presented the results of an experimental program involving 48 dynamic push–pull tests of concrete specimens placed on top of each other. All specimens were subjected to dynamic cyclic displacement loading with varying rates (2, 5, 10, and 20 mm/s), varying normal stresses (0 and 0.25 MPa), and varying surface roughness (as-cast or smooth, wire-brushed or medium rough, and raked or rough). From this study, the following conclusions are drawn:

1. Concerning the concrete surface condition after the test, surface deterioration was higher on rough surfaces than on smooth surfaces.
2. The most common friction phenomena related to the load–displacement hysteresis loop and load-velocity hysteresis loop (velocity effect, cycling effect, breakaway effect, non-reversibility, presliding displacement) were observed across all dynamic push–pull tests.
3. With time, the vertical load behavior depends on the surface roughness; on rough surfaces, a noticeable decrease in the vertical load was observed compared with the smooth surface. This trend confirms that the degradation on rough surfaces is higher than that on smooth surfaces, leading to a decrease in the recorded vertical load.
4. The vertical load time history depends on the test rate on rough surfaces. The vertical load decreases noticeably at higher loading rates.
5. The static and kinetic friction coefficients, effective stiffness, and effective damping decrease with increasing loading rates.
6. Increasing the normal stress increases the friction force, thus increasing the effective stiffness and decreasing the effective damping for all surface roughness types.

The study of concrete-to-concrete friction is essential; quantifying the friction and taking it into account is necessary to design composite structures and concrete members subjected to shear. ACI 318, AASHTO, CEB-fib model code, and Eurocode all provide shear design equations that consider the coefficient of friction. These codes give the coefficients of friction for different concrete surface conditions in the form of tables. However, all present design codes consider only the case of static friction and ignore the kinetic friction.

Therefore, based on the results of this study, the following is recommended:

1. The effect of loading rate and normal stress on the coefficient of friction and capacity of the interface should be considered in the provisions of design codes.
2. Rough surfaces that are widely used in construction practice should be favored in structures subjected to cyclic loading, taking into account the decrease in coefficient of friction with loading cycles.

Future research recommends expanding the current study to include higher stresses and lower and higher loading rates.

## Data Availability

The datasets used and analyzed during the current study are available from the corresponding author on reasonable request.
